# Redox Imbalance and Its Metabolic Consequences in Tick-Borne Diseases

**DOI:** 10.3389/fcimb.2022.870398

**Published:** 2022-07-22

**Authors:** Monika Groth, Elżbieta Skrzydlewska, Marta Dobrzyńska, Sławomir Pancewicz, Anna Moniuszko-Malinowska

**Affiliations:** ^1^ Department of Infectious Diseases and Neuroinfections, Medical University of Bialystok, Bialystok, Poland; ^2^ Department of Inorganic and Analytical Chemistry, Medical University of Bialystok, Bialystok, Poland

**Keywords:** redox, tick-borne, Lyme, oxidative stress, tick-borne encephalitis

## Abstract

One of the growing global health problems are vector-borne diseases, including tick-borne diseases. The most common tick-borne diseases include Lyme disease, tick-borne encephalitis, human granulocytic anaplasmosis, and babesiosis. Taking into account the metabolic effects in the patient’s body, tick-borne diseases are a significant problem from an epidemiological and clinical point of view. Inflammation and oxidative stress are key elements in the pathogenesis of infectious diseases, including tick-borne diseases. In consequence, this leads to oxidative modifications of the structure and function of phospholipids and proteins and results in qualitative and quantitative changes at the level of lipid mediators arising in both reactive oxygen species (ROS) and ROS enzyme–dependent reactions. These types of metabolic modifications affect the functioning of the cells and the host organism. Therefore, links between the severity of the disease state and redox imbalance and the level of phospholipid metabolites are being searched, hoping to find unambiguous diagnostic biomarkers. Assessment of molecular effects of oxidative stress may also enable the monitoring of the disease process and treatment efficacy.

## Introduction

According to the World Health Organization, infectious diseases pose a great threat to human health, accounting for about 17% of all deaths in the world in 2020 (out of 9.2 million deaths).Vector-borne diseases, including tick-borne diseases caused by pathogens such as bacteria (*Borrelia burgdorferi*, *B. garinii*, *B. afzelii*, and *Anaplasma phagocytophilum*) and viruses [tick-borne encephalitis (TBE) virus, Powassan virus, and Louping ill virus] and parasites such as *Babesia divergens*, *B. microti*, and *B. venatorum*, are an emerging global health issue (Kemenesi and Bányai, 2018; Boulanger et al., 2019; Madison-Antenucci et al., 2020). The most common vector-borne diseases in Europe include Lyme disease (LD), TBE, human granulocytic anaplasmosis (HGA), and babesiosis (Boulanger et al., 2019; Madison-Antenucci et al., 2020).

LD is the most common tick-borne illness in Europe and North America. It is a bacterial disease caused by a spirochete belonging to *B. burgdorferi* s.l. complex, which currently comprises at least 22 genospecies (Blazejak et al., 2018). Several of these genospecies have been found to be pathogenic to humans, including *B. afzelii*, *B. garinii*, *B. burgdorferi* sensu stricto (s.s.), *B. bavariensis*, and *B. spielmanii*. *B. afzelii* and *B. garinii* are the most common European circulating genospecies, followed by *B. burgdorferi* s.s. (Franke et al., 2013; Kim et al., 2020). In Europe, approximately 650,000–850,000 assumed new LD cases are reported each year, whereas, in the United States, approximately 476,000 people are affected by LD each year (Kugeler et al., 2021). These numbers underscore the large clinical burden associated with LD. Anaplasmosis, also known as HGA, is a tick-borne disease caused by the obligatory intracellular bacterium. Anaplasmosis is found worldwide, particularly in the northeastern United States, northern Europe, and Southeast Asia, although the geographic range of HGA cases appears to be expanding. The incidence of HGA cases in the USA has increased steadily since the disease became reportable from 348 cases in 2000 to 5,655 cases in 2019. The number of annually reported HGA cases in Europe is lower; however, there is insufficient epidemiological data as the disease is underdiagnosed or underreported (Matei et al., 2019; Eldaour et al., 2021). Ticks are vectors of a wide variety of viruses, also known as tick-borne viruses or tiboviruses (TBV). TBV are a diverse group of vertebrate viruses, which are classified into one DNA viral family, Asfarviridae, and eight RNA viral families: Flaviviridae, Orthomyxoviridae, Reoviridae, Rhabdoviridae, Nyamiviridae, Nairoviridae, Phenuiviridae, and Peribunyaviridae. Some TBV are pathogenic to humans and may pose a significant threat to health. TBE virus (TBEV) belonging to the Flaviviridae is the most recognized of the TBV (Kazimírová et al., 2017). Worldwide, approximately 10,000–15,000 cases of TBE are reported each year (Bogovic and Strle, 2015). Other tick-borne flaviviruses, such as Louping ill virus and Powassan virus, also produce encephalitis but are considered rare and their pathogenesis is not well studied, and literature data are limited (Kemenesi and Bányai, 2018).

It is worth mentioning that tick saliva proteins can also induce the alpha-gal syndrome, which is also known as red meat allergy. It is a novel form of anaphylaxis associated with IgE antibodies directed against the galactose-α-1,3-galactose (α-gal), an epitope that occurs also in mammalian food products (e.g., beef and pork) (Commins and Platts-Mills, 2013; Crispell et al., 2019). It has been demonstrated that α-gal is present in various tick species, including *Amblyomma americanum* and *Ixodes ricinus* (Sharma and Karim, 2021).

Tick-borne diseases are a significant problem both from an epidemiological and clinical point of view. These diseases are usually characterized by non-specific symptoms, which make the diagnosis challenging. In a rational diagnostic process, both epidemiological history and clinical picture as well as the results of laboratory tests should be taken into account (Riccardi et al., 2019; Madison-Antenucci et al., 2020). Erythema migrans (EM), an early form of LD, is diagnosed solely on the clinical picture, whereas the diagnosis of other forms of LD and other tick-borne diseases needs to be supported by the results of serological tests (LD, TBE, HGA, and babesiosis), molecular tests (LD, TBE, HGA, and babesiosis), and microscopic examination of peripheral blood smears (babesiosis and HGA) (Riccardi et al., 2019; Madison-Antenucci et al., 2020). Studies in recent years have shown that one of the most common metabolic disorders associated with tick-borne diseases is inflammation and redox imbalance, which leads to oxidative stress and oxidative modification of important structural and functional elements of the organism (Moniuszko-Malinowska et al., 2016; Jaganjac et al., 2020; Wawrzeniak et al., 2020). So far, the importance of oxidative stress has been proven in the pathogenesis of many diseases, including cardiovascular diseases, neurodegenerative diseases, cancer, and infectious diseases (Dominic et al., 2020; Jaganjac et al., 2020; Missiroli et al., 2020). Oxidative stress is accompanied by oxidative modifications of cellular components and body fluids, including proteins, lipids, and nucleic acids, which leads to complex metabolic changes resulting in pathophysiological disorders in the patient’s body and the intensification of the disease process (Moniuszko-Malinowska et al., 2016; Fedoce et al., 2018; Gęgotek and Skrzydlewska, 2019; Hawkins and Davies, 2019; ). Therefore, a thorough understanding of the pathophysiological changes, including the assessment of the level of pro-inflammatory factors, and the parameters of oxidative-antioxidant balance and their interactions in infectious diseases, including those transmitted by ticks, can contribute to proper diagnosis and differential diagnosis of various tick-borne diseases, but it may also enable the monitoring of the disease process and rational pharmacotherapy as well as the assessment of the effects of treatment.

## Oxidative Stress and Its Consequences in Infections

The proper functioning of each cell determines the proper functioning of the entire organism, and therefore, strict control of metabolic processes is required, including the transmission of signals from the environment, e.g., by pathogens. During infection, pathogens are detected, absorbed, and then phagocytosed by the host’s inflammatory cells, and signal processing of their presence in the human body can trigger both rapid and delayed responses. In the case of a quick reaction to changes, short-term modifications in the level/activity of the cells individual metabolic components occur. Late reaction is associated with the biosynthesis of new compounds, which is energy- and time-consuming, but ensures a long-lasting and effective response to the stimulus (Ingram, 2018).

**Figure 1:**
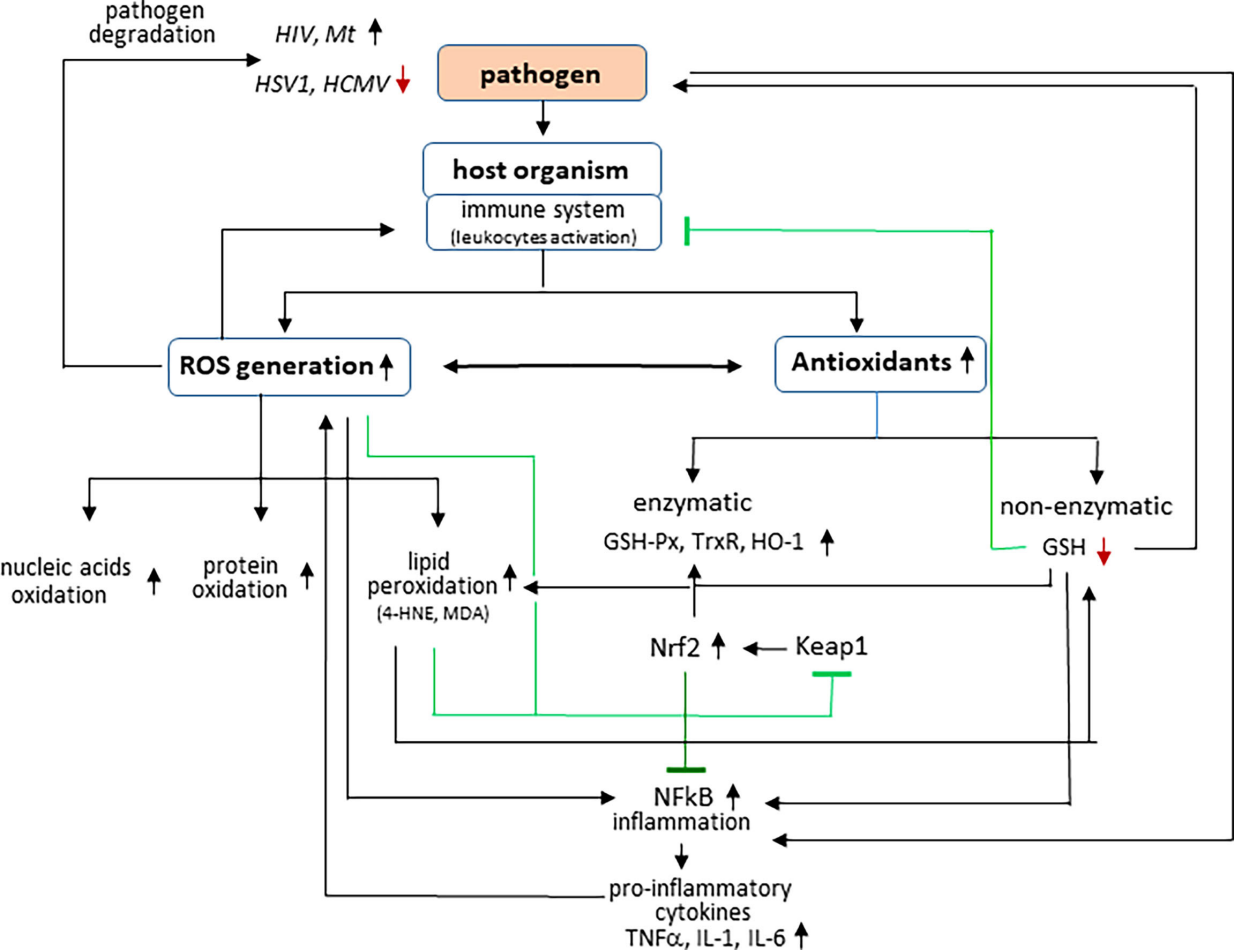
Effect of pathogens on interaction between ROS generation and inflammation in host organism.

During infection, host cells produce, receive, and react to signaling molecules that are formed in response to the presence of the pathogen in the body (Koo and Garg, 2019; Mullen et al., 2020). One of the basic conditions of the physiological state of the body is redox balance at the level of both cells and tissues, including body fluids (Trachootham et al., 2008; Lim and Leprivier, 2019). This is due to the fact that the physiological generation of reactive oxygen species (ROS) is kept at a low level, and their action is balanced by the complex of enzymatic and non-enzymatic antioxidants involved in maintaining redox homeostasis *in vivo* (Lee et al., 2019; Miyazawa et al., 2019). However, in response to the invasion of the pathogen, the host organism activates leukocytes, which produce large amounts of reactive oxygen and nitrogen species (ROS and RNS, respectively) as a consequence of enhanced activity of pro-oxidative enzymes responsible for the generation of ROS/RNS, which play a key role in the host’s immune defense against pathogens (**Figure 1**) (Griffiths et al., 2017). ROS and RNS are formed mainly in the mitochondrial respiratory chain and in the cytosol in the reaction catalyzed by nicotinamide adenine dinucleotide phosphate (NADPH) oxidase, which activity can be induced by pro-inflammatory cytokines such as interferon-γ (IFN-γ), interleukin-1 (IL-1), and IL-8 (Mullen et al., 2020) and at the level of biological fluids in reactions catalyzed by myeloperoxidase, xanthine oxidase, and nitric oxide synthase (Lee et al., 2019; Casas et al., 2020). Consequently, oxidants generated under infection conditions include superoxide anion radicals ( $O_{2}^{-}$ ), hydroxyl radicals (OH.), and hydrogen peroxide and nitric oxide (Zhang et al., 2014). The generated ROS modulate the signal transduction cascade and enhance the immune functions of lymphocytes (Belikov et al., 2015).

Infection is usually accompanied by changes in the effectiveness of endogenous antioxidant defense mechanisms, which consist of two distinct groups: enzymatic and non-enzymatic antioxidants. The most important antioxidant enzymes include superoxide dismutases (Cu, Zn-SOD; Mn-SOD), glutathione peroxidase (GSH-Px) and reductase (GSSG-R), catalase (CAT), and thioredoxin reductase, whereas non-enzymatic antioxidants include GSH, thioredoxin (Trx), ferritin, ceruloplasmin, ascorbic acid, and tocopherols (Ye et al., 2015). Together, the antioxidant mechanisms buffer the oxidants and maintain the redox balance. However, both viral and bacterial infections are usually accompanied by inflammation and a decrease in the effectiveness of endogenous antioxidant defense mechanisms. This situation favors the development of oxidative stress (Komaravelli and Casola, 2014; Shastri et al., 2018). Disruption of redox homeostasis by pathogens leads to the modifications of both cellular metabolism and intra- and extracellular signaling. Evidence of the formation of oxidative stress in the human body has been found in many infectious diseases, such as viral hepatitis (B, C, and D), AIDS, human papillomavirus (HPV) infection, and tuberculosis (Ivanov et al., 2017; Georgescu et al., 2018; Shastri et al., 2018). It is also believed that oxidative stress plays an important role in the course of flavivirus infections, including those caused by hepatitis C virus, dengue virus, Zika virus, Japanese encephalitis virus, West Nile virus, and Usutu virus (Zhang et al., 2019; Blázquez et al., 2021). Flaviviruses have been shown to induce oxidative stress in infected cells *in vivo* and *in vitro* (Gullberg et al., 2015)

Oxidative stress plays a double role in infectious diseases because ROS modify both the susceptibility and resistance of the host organism to infections (Ivanov et al., 2017). Moreover, because of their non-specific nature and unique reactivity, ROS become harmful to both the pathogen and the host (Pohanka, 2013). The overproduction of ROS promotes the degradation of pathogens and thus protects the body against their effects. However, it is known that elevated levels of ROS may increase cytotoxicity and damage to the host’s molecules/cells/organs, whereas their reduced levels may favor the survival and spread of pathogenic microorganisms (Ivanov et al., 2017; Novaes et al., 2019). The consequences are similar in both situations, but lowering the ROS level usually leads to higher host mortality (Novaes et al., 2019). It has even been suggested that ROS not only help the host organism to cope with pathogenic microbes but also take part in inter-bacterial competition that protects the human body against multi-pathogenic infection (Nathan and Cunningham-Bussel, 2013). On the other hand, some microorganisms such as *Trypanosoma cruzi*, human immunodeficiency virus (HIV), or *Mycobacterium tuberculosis* have been shown to survive best and even thrive under oxidative conditions (Kashou and Agarwal, 2011; Paiva et al., 2018).

The most important fact is that the oxidative stress that occurs as a result of the invasion of the pathogen is dangerous for the functioning of the host organism, because the overproduction of ROS promotes their involvement in oxidative structural modifications of the basic components of cells/biological fluids, including DNA, lipids, and proteins. It can alter their functionality and promote inflammation, which is crucial for the host’s immune response. Viral infections cause both direct cytopathogenic effects and an excessive inflammatory response in the infected host. Therefore, two cooperating transcription factors play a particularly important role in the cellular response to pathogen invasion: NF-κB, responsible for the transcription of pro-inflammatory factors, and Nrf2, responsible for the transcription of cytoprotective, including antioxidant proteins (Ingram, 2018). Recent studies have shown that oxidative stress conditions promote activation of the redox-sensitive NF-κB signaling pathway, which not only induces the expression of cell adhesion receptors, pro-inflammatory cytokines, and chemokines involved in the production and development of ROS and the maintenance of inflammation but is also closely involved in virus replication and in host cell proliferationa/poptosis (Checconi et al., 2019; Kerstholt et al., 2020; Mullen et al., 2020). Activation of NF-κB induces pro-inflammatory genes, including those encoding tumor necrosis factor (TNF-α), IL-1, and IL-6 (Ingram, 2018; Lingappan, 2018; Fraternale et al., 2021). However, the transcriptional and signaling activity of NF-κB depends on interaction with the basic non-enzymatic cellular antioxidant—GSH, which modifies the structure of NF-κB through glutathionylation, inhibiting its pro-inflammatory effects. In fact, protein glutathionylation is the main redox immune mechanism that affects the function of not only NF-κB but also of proteins such as signal transducer and activator of transcription 3 (STAT3), protein kinase A (PKA) , TNF receptor-associated factor (TRAF3) and TNF Receptor Associated Factor 6 (TRAF6) (Mullen et al., 2020).

GSH as the basic non-enzymatic cytosolic antioxidant, co-substrate of GSH-Px, supports its antioxidant activity by preventing peroxidation of membrane phospholipids and, consequently, disruption of the structure and function of biological membranes (Schmitt et al., 2015; Angelova et al. 2021). Inhibition of lipid peroxidation simultaneously decreases the level of low–molecular weight electrophilic aldehydes, such as malondialdehyde (MDA) and 4-hydroxynonenal (4-HNE), which, like lowering the level of ROS, reduces the likelihood of modification of nucleophilic elements of protein molecules, such as, for example, Keap1—cytosolic inhibitor of the transcription factor Nrf2, which regulates the transcription of genes, including those encoding proteins involved in the maintenance of redox balance, detoxification, repair of macromolecular damage, and metabolic balance (Cuadrado et al., 2019). Under physiological conditions, Nrf2 forms a complex with Keap1, which directs the transcription factor to ubiquitination and proteasomal degradation (Ma et al., 2015). However, under oxidative conditions, both ROS and other electrophiles, including lipid peroxidation products, reacting with the critical Keap1 cysteine residues inactivate this inhibitor, resulting in Nrf2 activation and increased genes transcription (Ma, 2013).

Nrf2 is thought to protect cells from stress-induced death, and therefore, Nrf2 has been suggested to be the major regulator of tissue damage during infection (Soares and Ribeiro, 2015). Furthermore, Nrf2 is known to play a role in both the induction and resolution of inflammation (Ma et al., 2015) by repressing pro-inflammatory genes such as IL-6 and IL-1β (Silva-Palacios et al., 2018). The analysis of single-cell RNA sequencing showed that the signature of the Nrf2 antioxidant genes correlates with resistance to HSV1 infection (Wyler et al., 2019). In addition, it was shown that, in biopsies from patients with COVID-19, inhibition of Nrf2-dependent genes expression is observed, and the use of Nrf2 agonists, 4-OI and DMF, reduces SARS-CoV2 replication, and a similar situation was indicated with other viruses, including Herpes Simplex-1/-2 (HSV-1 and HSV-2), vaccinia virus (VACV), and Zika virus (ZIKV) (Olagnier et al., 2020).

In inflammatory conditions, Nrf2 activation restores redox homeostasis by upregulating essential antioxidants such as heme oxygenase 1 (HO-1), thioredoxin, thioredoxin reductase, GSH-Px, and peroxiredoxin, which protect the host against oxidative stress (Ingram, 2018; Lee, 2018). It is known that increasing the level of HO-1, the basic product of Nrf2 transcriptional activity, promotes effective cellular immunity to numerous pathogens (Huang et al., 2017). This explains why responses to introduction of the pathogen to the human body and, consequently, increased ROS production are usually accompanied by an overproduction of antioxidants, including enzymatic antioxidants (Ingram, 2018; Lee, 2018). Nrf2 is closely related to the immune response to different types of antigens and convergent signaling during pathogen recognition (Battino et al., 2018; Mohan and Gupta, 2018; Olagnier et al., 2018). However, Nrf2 may show an equivocal response that either favors advanced infection or induces bactericidal activity of defense cells (Deramaudt et al., 2013; Shah et al., 2018; Cuadrado et al., 2019). The molecular mechanisms of immunity modeled by Nrf2 in response to stress, including that from intracellular infections, may vary with the species of pathogen and its virulence, as well as with the immune response of the host. Moreover, it has been shown that there are viruses capable of redirecting the Nrf2 pathway, allowing infection to progress (Lee, 2018). This increases the transcription of cytoprotective proteins and anti-inflammatory effects by reducing the transcription of NF-κB target genes, including TNFα, IL-1β, and IL-6 (Fraternale et al., 2021).

GSH is an antioxidant, which inhibits the production of most inflammatory cytokines activated by ROS, but at the same time, this tripeptide is required to restore and/or maintain adequate levels of IFN-γ production by cells presenting antigen and is essential for an effective immune response against intracellular pathogens (Murata et al., 2002a). Thus, lowering GSH level may promote viral replication/multiplication also by attenuating the host’s antiviral immune response. Abnormal antigen processing and decreased IL-12 secretion have been found to correlate with GSH deficiency in antigen presenting cells, which promotes Th1/Th2 bias toward Th2 (Amatore et al., 2019). However, by increasing the level of intracellular GSH, it is possible to restore a balanced Th1/Th2 immune response during viral infection or to enhance the Th1 immune response to antigens (Murata et al., 2002b; Fraternale et al., 2010). It has also been shown that several genes important for antiviral activity, including the antiviral proteins Oas and Mx2, require the presence of GSH (Murata et al., 2002b). This supports suggestions that GSH can inhibit viral infections and that viral infections induce the appearance of glutathionylated thioredoxin and PRDX family (Khomich et al., 2018; Fraternale et al., 2021). Therefore, reducing GSH level as a result of oxidative stress may be a way to attenuate the host’s antiviral response by weakening GSH-dependent metabolic antiviral pathways.

In response to pathogen invasion, the host organism activates leukocytes, which produce large amounts of reactive oxygen species (ROS), which are harmful to both the pathogen and the host. Some microorganisms such as herpes simplex virus type 1 (HSV1) and human cytomegalovirus (HCMV) are degraded in oxidative conditions; however, other pathogens, such as human immunodeficiency virus (HIV) or *Mycobacterium tuberculosis* (Mt) have been shown to survive best under oxidative conditions. Infections are also accompanied by changes in the effectiveness of antioxidants among which the activity of, e.g., glutathione peroxidase (GSH-Px), thioredoxin reductase (Trx-R), and heme oxygenase (HO-1) is increased, whereas the level of glutathione (GSH) is reduced. In addition, ROS promote, among others, oxidative modifications of macromolecular compounds, including lipid peroxidation with the generation of among others 4-hydroxynonenal (4-HNE) and malondialdehyde (MDA). Both ROS and lipid peroxidation products modify the structure of proteins, which, *inter alia*, leads to the activation of transcription factors: nuclear factor erythroid 2 (Nrf2) (by modifying its inhibitor, Keap1) and nuclear factor kappa light-chain enhancer of activated B cells (NF-κB) inducing pro-inflammatory genes, including tumor necrosis factor (TNF-α) and interleukins: IL-1 and IL-6.

### Lipid Metabolism in Infections

Taking into account the fact that viral, bacterial, or parasitic infections are usually accompanied by oxidative stress, the increased level of ROS promotes metabolism of lipids in the cells/organism of the host, whose metabolites are considered mediators of inflammation (Pohanka, 2013; Koriem, 2017). Lipid metabolism is controlled by ROS and/or enzymes, mainly phospholipases, cyclooxygenases (COXs), and lipoxygenases (LOXs), the activity of which is increased under oxidative stress (Ayala et al., 2014). The resulting lipid mediators coordinate both the initiation of the immune response against pathogens and the resolution of the infection (Tam, 2013). It has been shown that the proportions of pro-inflammatory and anti-inflammatory lipid mediators can determine the pathogenicity of specific microorganisms (Tam, 2013). It has been found, *inter alia*, that treatment of ferrets infected with influenza virus, a sphingosine-1-phosphate (S1P) receptor agonist, inhibits the production of cytokines and chemokines during infection, which indicates the participation of lipid mediators in the regulation of inflammation during infection as well as the prevention of pathogens action (Teijaro et al., 2014).

**Figure 2:**
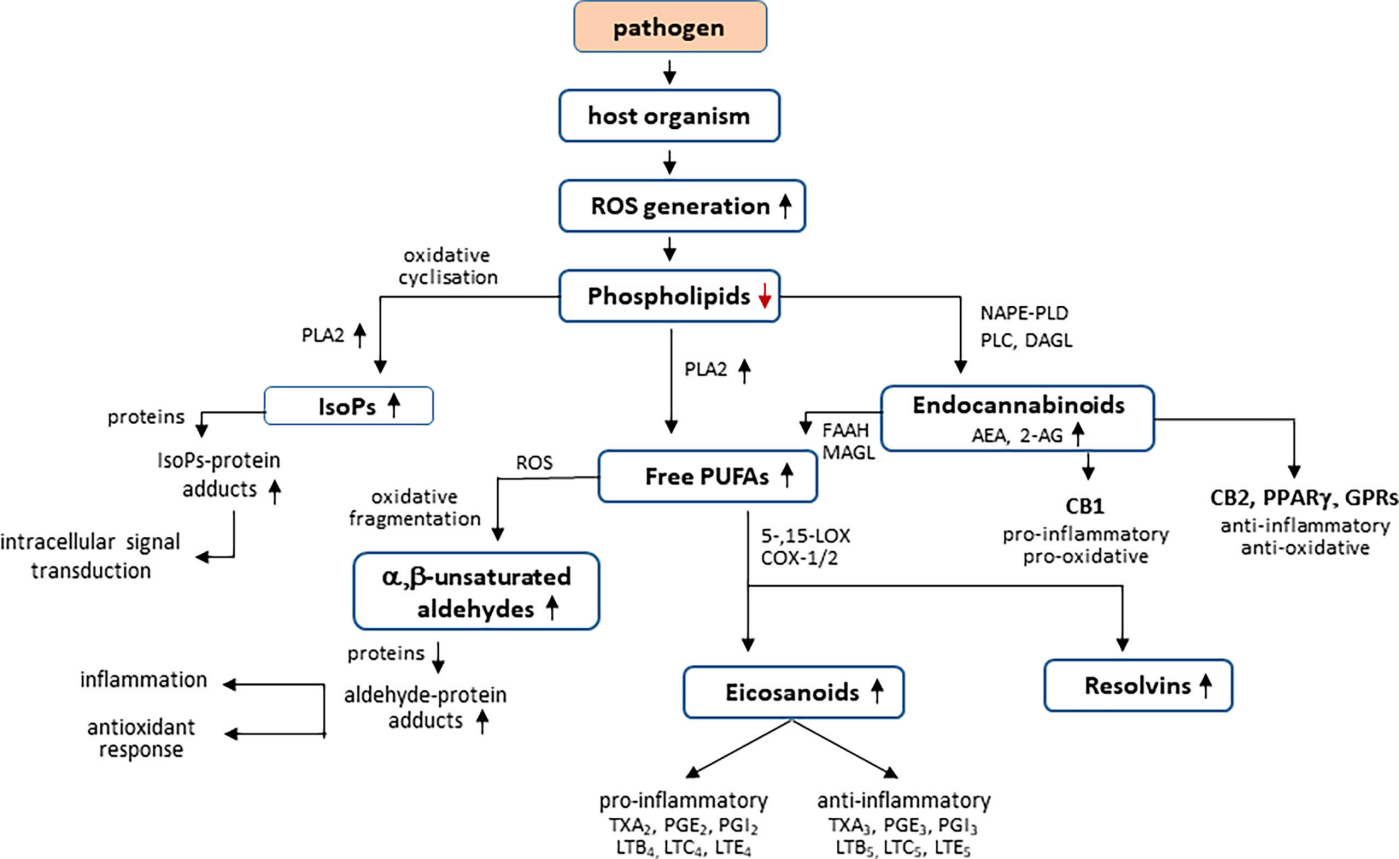
Effect of pathogens on phospholipid metabolism in host organism.

Among lipids, an important role is played by phospholipids, which are structural elements of biological membranes, including cell membranes. The phospholipid bilayer is, among others, a platform for proteins involved in cell signaling influencing intercellular communication, regulation of gene expression and immune response (Chaurio et al., 2009; Muallem et al., 2017; Lydic and Goo, 2018). Moreover, phospholipids play an important role in the regulation of metabolic processes including cell proliferation, migration, and apoptosis (Chaurio et al., 2009). On the other hand, under oxidative conditions, ROS interact with membrane phospholipids, especially those containing polyunsaturated fatty acids (PUFAs), especially arachidonic, linolenic, eicosapentaenoic, and docosahexaenoic acids, which are particularly susceptible to oxidative modifications (Ayala et al., 2014). Non-enzymatic transformations are based on free radical chain reactions, initiated mainly by hydroxyl or hydroperoxide radicals, arising endogenously as a result of cellular metabolism (i.e., respiratory chain reactions, reactions catalyzed by oxidases: NADPH and xanthine) and generated by the action of pathogens (Su et al., 2019). The conversion of lipid hydroperoxides is based on their intramolecular cyclization or fragmentation. As a result of cyclization, prostaglandin derivatives containing a characteristic five-membered prostane ring are formed, including isoprostanes (mainly F2-isoprostanes) and neuroprostanes (mainly F4-neuroprostanes) (**Figure 2**) (Ayala et al., 2014; Miller et al., 2014). The prostaglandin derivatives are cleaved from phospholipid structures by phospholipase A2 (PLA2) (Miller et al., 2014). Prostaglandin derivatives participate in the pathogenesis of many diseases, including infectious diseases (Dennis and Norris, 2015). This is especially true of F2-isoprostanes, which are present in detectable amounts in all tissues of the body and are therefore considered reliable biomarkers of oxidative stress and peroxidation of phospholipids, and their concentration is not related to the amount of fat consumed in the diet (Comporti et al., 2004). The best known isomer of F2-isoprostanes is 8-isoprostaglandin F2α (8-iso-PGF2α), which was used to assess oxidative stress in the course of infectious diseases such as pneumonia, malaria, and COVID-19 (Muhammad et al., 2020; Zheng et al., 2021).

An alternative mechanism for the conversion of lipid hydroperoxides is the fragmentation of their hydrocarbon chains to form α,β-unsaturated, reactive aldehydes, including 4-HNE and MDA (Dalleau et al., 2013; Galano et al., 2017; Ahmed et al., 2020). Because of their electrophilic nature, low–molecular weight aldehydes have the ability to form adducts with compounds containing nucleophilic centers, such as proteins, DNA, and phospholipids, modifying their structure and functions, which leads to, among others, disruption of the integrity of biological membranes or inactivation of the biological activity of proteins (Nicolson and Ash, 2017; Pohl and Jovanovic, 2019). This applies to situations where lipid peroxidation is significantly increased and the concentration of aldehydes increases even several times. In addition, the formed aldehydes easily diffuse through biological membranes and modify proteins even in distant places, both in the cellular and extracellular environment, and in biological fluids, influencing the signal transmission process (Dalleau et al., 2013; Gęgotek and Skrzydlewska, 2019). Increased level of 4-HNE and MDA has been observed in tissues and body fluids in the course of infectious diseases (Rajopadhye, 2017; Rajashekaraiah et al., 2021). Aldehydes, particularly extremely reactive 4-HNE, generated during lipid peroxidation interact with proteins, causing changes in the structure of both phospholipids and proteins, and consequently disrupt the functioning of the blood–brain barrier, increasing its permeability (Enciu et al., 2013).

The process of ROS-dependent PUFA oxidation is supported by the oxidative action of two main groups of enzymes: COXs and LOXs with the necessary participation of phospholipases, mainly PLA2. This phospholipase releases the products of oxidative cyclization of PUFAs from the phospholipid structure, and by hydrolyzing membrane phospholipids, it provides free fatty acids (Joshi et al., 2016). However, LOXs and COXs, on the other hand, by catalyzing the attachment of the oxygen molecule to the fatty acid chain, increase the amount of lipid hydroperoxides formed with participation of ROS, which are further oxidized with the generation of reactive aldehydes (Gęgotek and Skrzydlewska, 2019; Su et al., 2019).

As a result of enzymatic transformations of phospholipids, a group of lipid mediators important for cellular metabolism is formed—endocannabinoids, which are ester, ether, and amide derivatives of fatty acids, especially arachidonic acid (Lu and Mackie, 2016; Lu and Mackie, 2021). Endocannabinoids are part of the endocannabinoid system (ECS), which also includes cannabinoid receptors (CB1/2) and enzymes involved in the biosynthesis and degradation of endocannabinoids (Lu and Mackie, 2021). The best known endocannabinoids are N-arachidonylethanolamide (AEA) and 2-arachidonyl glycerol (2-AG). AEA is biosynthesized from phosphatidylethanolamines and phosphatidylcholines located in the outer layer of the cell membrane in reactions mediated mainly not only by N-acyltransferase and NAPE phospholipase D (PLD) but also by phospholipase C (PLC), PLA2, and α/β-hydrolase 4 (Apostu et al., 2019). However, 2AG biosynthesis begins from the hydrolysis of membrane phospholipids (phosphatidylinositol, phosphatidylcholine, and phosphatidylethanolamine) catalyzed by PLC to form 1,2-diacylglycerol (DAG), which undergoes further hydrolysis catalyzed by diacylglycerol lipase (DAGL) to 2-AG (Lu and Mackie, 2016). Endocannabinoids act as agonists of not only G protein–coupled receptors, including cannabinoid receptors (CB1/2), but also TRPV1 and GPR receptors (Lu and Mackie, 2016; Lucaciu et al., 2021). Both cannabinoid receptors are involved in the regulation of redox balance and inflammation, including CB1 by increasing the production of ROS and TNF-α, which is observed, among others, in viral diseases; whereas the CB2 receptor, expressed mainly in cells of the immune system and tissues (Yao and Mackie, 2009), by decreasing the production of ROS and TNF-α, not only reduces both oxidative stress and inflammation (Han et al., 2009; Cinar et al., 2022) but also regulates mitogen-activated protein kinases (MAPKs), and some MAPKs also stimulate phospholipases C and A2 (Correa et al., 2009; Han et al., 2015; Tahamtan et al., 2015).

Lipid metabolism is controlled by ROS and enzymes including phospholipase 2 (PLA2), cyclooxygenases 1/2 (COX1/2), and 5-,15-lipoxygenases (5-,15-LOX). ROS interact with membrane phospholipids, especially those containing polyunsaturated fatty acids (PUFAs) with products generation of intramolecular cyclization [isoprostanes (IsoPs)] or fragmentation (a,b-unsaturated aldehydes). Both free PUFAs and IsoPs are cleaved from phospholipid structures by PLA2. However, enzymatic phospholipid metabolism by NAPE-PLD (N-acyl phosphatidylethanolamine–specific phospholipase D), PLC (phospholipase C), and DAGL (diacylglycerol lipase) leads to endocannabinoids [N-arachidonylethanolamide (AEA) and 2-arachidonyl glycerol (2-AG)] formation, which are degraded to free PUFAs by fatty acid amidohydrolase (FAAH) and monoacylglycerol lipase (MAGL). Endocannabinoids act as agonists/antagonists of cannabinoid receptors (CB1/2) as well as the peroxisome proliferator–activated receptor g (PPARg) and G protein–coupled receptors (GPRs). However, as a result of enzymatic metabolism of the PUFAs, generated from phospholipids and endocannabinoids, anti-inflammatory or pro-inflammatory eicosanoids [thromboxanes (Tx), prostaglandins (PG), and leukotrienes (LT)], and resolvins are formed.

Because of the involvement of the ECS in the regulation of oxidative stress and inflammation, endocannabinoids, by activating CB1/2 receptors, influence the course of viral infections. It has been found that induction of CB1 receptors during viral infections in neuronal cells can activate the ERK-phosphorylating MAPK cascade and reduce Ca2^+^ ion concentrations in cells, which lowers nitric oxide (NO) production and pro-inflammatory mediators, which are essential for the development of host responses to viral infections (Reiss, 2010). It has also been suggested that endocannabinoids and CB1 receptors are part of a pathway involved in the development of hepatocyte glucose metabolism disorders due to HCV infection (Sun et al., 2014). Many viruses alter host cell physiology and enhance certain metabolic pathways to increase the rate of viral replication (Fontaine et al., 2014; Sanchez and Lagunoff, 2015; Heaton and Randall, 2011). However, it has also been observed that, in the case of infection with Sindbis virus, the stimulation of the CB1 receptor and inactivation of alternative cellular pathways that reduce the available resources in the cell and the inhibition of virus replication are observed (Rodriguez et al., 2021). The ECS is involved in all internal interactions of the human body, including the functioning of the immune system (Pacher, 2006; Lu and Mackie, 2016; Apostu et al., 2019). It has been suggested that its activation may be very beneficial, e.g., in inhibiting COVID-19 by the reduction of viral replication and the level of pro-inflammatory cytokines such as IL-2, IL-4, IL-6, IL-12, TNF-α, and IFN-γ (Nichols and Kaplan, 2020; Lucaciu et al., 2021). Moreover, an increase in endocannabinoid levels may lead to an increase in anti-inflammatory cytokines such as IL-10 (Lucaciu et al., 2021). Thus, activation of cannabinoid receptors is important for the pathogenesis of viruses affecting the CNS, such as HHV, HIV, VSV, BDV, measles, mumps, rabies, enteroviruses, La Crosse encephalitis virus, and lymphocytic meningitis virus (Tahamtan et al., 2015). On the other hand, because the CB2 receptor is present in large amounts in immune cells, activation of CB2 receptors exerts a protective effect by suppressing inflammation, oxidative stress, and beneficial regulation of the immune system to viral and bacterial infections (Rom and Persidsky, 2013; He et al., 2019). Moreover, it is believed that, in most cases, activation of cannabinoid receptors increases the progression of infectious diseases by modulating the host’s immune response (Reiss, 2010). In viral infections in which the host’s inflammatory response is immunopathogenic, activation of the receptors is beneficial for the control of disease development, progression, and pathology (Reiss, 2010). Activation of CB2 receptors also involves the modulation of numerous immune, inflammatory, and redox signaling pathways such as SIRT1/PGC-1α, AMPK/CREB, MAPK/ERK, and Nrf2/Keap1/HO-1 (Correa et al., 2009; Hashiesh et al., 2020). In addition, endocannabinoids, by activating PPAR family receptors, including PPAR-γ, which suppress inflammatory responses, enhance the host’s response against respiratory viral infections and inhibit the replication of many viruses, including influenza A (O’Sullivan, 2016).

In contrast, unbound with receptors, anandamide and 2AG are metabolized by hydrolysis and oxidation. Hydrolysis to arachidonic acid is catalyzed by intracellular serine amide hydrolases fatty acids (FAAH and MAGL) (Iannotti et al., 2016). Moreover, because AEA is structurally similar to PUFAs, it can be oxidized by LOXs and COX-2 and under the influence of activity of cytochrome P450 (Iannotti et al., 2016). AEA is oxidized by LOX-12, LOX-15, and LOX-5, respectively, to 12-HETE-EA, 15-HETE-EA, and 11-HETE-EA (Alhouayek and Muccioli, 2014; Dennis and Norris, 2015). In contrast, COX-2 oxidizes AEA to prostaglandin-H2-ethanolamide (PGH2-EA), which is hydrolyzed to prostaglandin derivatives acting by activation of GPCR and PPAR receptors, the activation of which affects both oxidative stress and inflammation (Alhouayek and Muccioli, 2014).

As a result of enzymatic degradation of endocannabinoids, especially anandamide, as well as enzymatic metabolism of phospholipids, free arachidonic acid is formed, which is then metabolized by COXs, LOXs, and CYP450. Under the conditions of oxidative stress accompanying infections, ROS-dependent genes, including COX2, are upregulated (Ivanov et al., 2017). As a result of the abovementioned enzymes, a heterogeneous group of PUFAs metabolites is formed—eicosanoids, including prostaglandins, thromboxanes, leukotrienes, hydroxyeicosatetraenoic acids, and lipoxins (Wang et al., 2019). Prostaglandins and leukotrienes are the main mediators of the inflammatory response in an experimental *M. tuberculosis* infection, which specifically induces the production of PGE2 and LTB4 in the lungs (Sorgi et al., 2020). In contrast, 5-LOX catalyzes the sequence of reactions to form LTA4, which can be hydrolyzed to LTB4 by LTA4 hydrolase or conjugated to GSH to form LTC4 catalyzed by LTC4 synthase (Sorgi et al., 2020). However, both isoforms of COX (COX1/2) metabolize arachidonic acid to prostaglandins (PGE2, PGD2, PGF2, and PGI2) or thromboxanes (TXA2) by specific synthases in various cells (Ricciotti and FitzGerald, 2011). Eicosanoids exhibit multidirectional physiological effects, including mainly pro-inflammatory effects generated by prostaglandins, thromboxanes, leukotrienes, and HETE derivatives, whose concentrations are elevated in infectious diseases (Dennis and Norris, 2015). In turn, E and D series resolvins and protectins produced from EPA and DHA play an important role in reducing inflammation (Dennis and Norris, 2015; Serhan et al., 2015). It has been suggested that eicosanoids, regulating the production of antibodies and cytokines of the immune system, as well as cell differentiation and proliferation, play a key role in modulating physiological and pathophysiological processes (Dennis and Norris, 2015), and only the analysis of all arachidonic acid metabolites may indicate the metabolic consequences of infection. It is known that controlling the immune response to infection involves complex molecular signaling networks with coordinated and often opposing actions. In addition, eicosanoids are involved in modulating both the pro-inflammatory and anti-inflammatory responses in the body through the activation of various receptors (Dennis and Norris, 2015).

In addition, it is known that different eicosanoids show different directions of action, which also depend on the type of pathogen. It has been shown that, in the case of *M. tuberculosis* infection, PGE2 inhibits necrotic death of macrophages, which results in the enhancement of host resistance to pathogens, and the level of TNF-α resulting from the balance between LTB4 and LXA4 is important for infection control (Mayer-Barber and Sher, 2015). In contrast, in the case of infection with influenza A virus, PGE2 weakens the host’s immunity by inhibiting the presentation of the macrophage antigen and the resistance of T55 cells (Coulombe et al., 2014). The results of recent years suggest that, in the pathogenesis of tuberculosis, the balance between PGE2 and LTB4 is important, which prevents severe inflammation (Sorgi et al., 2020). However, the products of the transformation of omega-3 PUFAs (ω-3 PUFAs) are resolvins, including the E series formed from eicosapentaenoic acid (EPA) under the influence of COX-2 and 5-LO, and the D series resolvins formed from docosahexaenoic acid (DHA), under the influence of 15-LO (Serhan and Levy, 2018). It has also been shown that resolvins, RvD1 and RvD5, act in synergy with protectins and enhance bacterial phagocytosis in human macrophages, reducing the need for antibiotics to remove microbes from mice (Chiang et al., 2012). This suggests that eicosanoid-associated signaling has implications for the organism’s bactericides response in the event of infection. In addition, lipoxins and their DHA metabolite counterparts, when used with bactericides, are believed to increase microbial clearance, at least partly due to the action of prostaglandins and leukotrienes. On the other hand, in the body of patients infected with the influenza virus and characterized by an increased level of cytokines and chemokines, a significantly higher percentage of eicosanoids from the LOX and CYP pathways is observed, including the following acids: EPA and DHA, such as PGE2, LTE4, and 17-hydroxy-DHA, the level of which was elevated in nasopharyngeal lavage compared to patients with low and moderate cytokine production (Ramon et al., 2014). This indicates that some eicosanoids are lipid mediators that favor the neutralization and elimination of pathogens, but the exact mechanism of their action is not entirely clear.

### Redox Homeostasis in Tick Hematophagy

Ticks are hematophagous ectoparasites infesting a wide range of vertebrate hosts, including humans and animals. During hematophagy, as a result of hemoglobin hydrolysis, iron ions are released, which participate in the generation of hydroxyl radicals, which are strong oxidants that can cause oxidative modification of macromolecules, including proteins and lipids, and promote mutation, cellular dysfunction, and cell death. To avoid damage caused by the interaction of ROS with cellular structures, blood-ingesting arthropods have developed molecular mechanisms that prevent these reactions from eliciting and reduce the resulting oxidative stress. One of the defense systems against heme toxicity is the aggregation of the released heme molecules in cell organelles. An example is the tick *Rhipicephalus microplus*, which is accumulates heme in organelles called hemosomes. Another mechanism that prevents heme from reacting with cellular particles is through heme transfer proteins, which have been well characterized in *Rhipicephalus* ticks. These proteins are also transporters and heme reservoirs, which is essential for the development of the tick embryo. Another defense mechanism is the degradation of the heme by heme oxygenase (HO), an enzyme that catalyzes the oxidation of the heme to biliverdin, carbon monoxide (CO), and Fe^2 +^. On the other hand, in *R. microplus*, the activity of catalase and GST is associated with oxidative stress and lipid peroxidation observed during the development of the egg and larvae and the process of hematophagy. It was shown that catalase is involved in control of hydrogen peroxide levels in *R. microplus* ticks during blood feeding. Furthermore, in *Ixodes ricinus* ticks, more sulfotransferase transcripts were detected after blood feeding. Importantly, GST has already been used as a protective vaccine antigen against ticks in multiple studies, which shows its potential in possible composition of an efficient vaccine (Sabadin et al., 2019).

### Tick-Pathogen Interactions and Redox Metabolism

Ticks have a proactive antioxidant system and a high tolerance to oxidizing agents, which is crucial for their survival. One of the preventive mechanisms is the antioxidant defense system associated with selenium (Se), which is essential for redox homeostasis, and recent studies suggest an important role for selenoproteins in protecting the tick microbiome from high oxidative stress (Kumar et al., 2016). Tick selenoproteins not only regulate oxidative and endoplasmic reticulum stress during prolonged tick feeding on mammalian hosts but also take part in pathogen colonization and maintenance of microbiota (Kumar et al., 2019). The presence of the vector-borne pathogen may alter the expression of the genes in the vector. The survival of tick borne pathogens has been shown to be related to the manipulation of the selenium-related antioxidant defense system, which contains a full complement of selenoproteins and other antioxidants. Tick-borne pathogens, including *Borrelia burgdorferi* and *Anaplasma phagocytophilum*, upregulate different sets of genes in the tick vector and their mammalian hosts (Iyer et al., 2015; Villar et al., 2015), suggesting differences between intracellular and extracellular pathogens. However, the exact differences between the pathogens and their relevance to the pathogenesis of the disease remain unclear. For example, it has been suggested that selenogene K may play an essential role in B. burgdorferi colonization of tick vectors and survival within the tick host (Kumar et al., 2019) Furthermore, it has been demonstrated that selenium level in the immature and mature tick stages increases after blood feeding, but selenium level decreases in *Amblyomma maculatum* ticks after SECIS binding protein 2 (SBP2) and putative selenoprotein P (SELENOP) knockdown. In addition, the SBP2 and SELENOP silencing reduces transovarial (vertical) transmission of *R. pakeri* to tick eggs and egg hatching, which is a possible novel control target for tick-borne pathogens and their vectors (Budachetri et al., 2017a). Another selenoprotein with vital functional role in maintaining the tick-associated bacterial community is thioredoxin reductase. Depletion of TrxR reduces viability of the microbiome within the tick tissues and negatively affects the bacterial load probably through perturbation of redox homeostasis. In salivary glands of knockdown ticks, the antioxidant genes *MnSOD*, *Cu/ZnSOD*, and *SelM* were significantly downregulated. (Budachetri and Karim, 2015). Transcription of selenogenes has been also shown to be upregulated in *R. parkeri-*infected *A. maculatum* ticks. Silencing of expression of three proteins: selenoprotein M, selenoprotein O, and selenoprotein S, leads to impaired *R. parkeri* colonization in the tick and is associated with compensatory response from other selenogenes. In selenogene-knockdown ticks, the oxidative stress levels and endoplasmic reticulum stress inside the ticks increase (Budachetri et al., 2018). Interestingly, however, one of the studies revealed that knockout of Selenoprotein K and Selenoprotein M in *A. maculatum* ticks does not decrease the salivary antioxidant activity. On the contrary, total antioxidant capacity in tick saliva was higher in selenoprotein knockouts, which suggests activation of compensatory mechanisms involved in eliminating ROS (Adamson et al., 2014)

Maintenance of redox balance seems to be an essential part of vector competence. In *A. phagocytophilum–*infected *Dermacentor variabilis* ticks with knockdown of genes encoding putative GSH S-transferase (GST), salivary selenoprotein M (SelM), H+ transporting lysosomal vacuolar proton pump (vATPase), and subolesin, there were no salivary gland infections, which suggests that, perhaps, silencing these genes makes the vectors incompetent of transmitting the pathogen. It was also suggested that *A. marginale* may increase the expression of SelM and GST as a way to reduce the oxidative stress caused by pathogen infection and thereby increase pathogen multiplication in tick cells (Kocan et al., 2009). There is evidence that mitochondrial ROS production pathways limit *A. phagocytophilum* infection and multiplication in tick cells. It has been proposed that tick cells reduce the antioxidant defenses to favor ROS accumulation and limit infection with this intracellular pathogen. *A. phagocytophilum* utilizes common mechanisms for infection of tick vectors and vertebrate hosts; however, there may be differences in ROS response to the infection between tick and human cells (Alberdi et al., 2019). It seems that redox homeostasis is of vital importance to the survival of the pathogen within the tick (Karim et al., 2021). It has been established that SODs are essential defense mechanisms against the damage caused by ROS within the tick. They are also important for regulation of bacterial communities inside the tick vectors and aid the colonization of certain pathogens, e.g., *R. parkeri* in the vectors. Knockdown of these enzymes increases total oxidative stress in ticks. It has been indicated that tick-associated pathogens rely on maintaining redox homeostasis while also producing ROS within the tick cells and using them as signaling molecules for regulation of bacterial density (Crispell et al., 2016). Silencing of Cu,Zn-SOD and/or Mn-SOD leads not only to elevated level of oxidative stress but also to the reduction of the *R. parkeri* load (Crispell et al., 2016; Karim et al., 2021). Catalase gene expression in salivary glands, midgut, and ovarian tissues of *Amblyomma maculatum* significantly increases during *R. parkeri* infection. Furthermore, silencing the other primary antioxidant enzyme catalase not only interferes with reduced microbial load but negatively affects tick fecundity and transovarial transmission of *R. parkeri.* This suggests that catalase is essential for rickettsial colonization of the tick vector and transmission of the pathogen (Kumar et al., 2016; Budachetri et al., 2017b; Karim et al., 2021). Moreover, it has also been suggested that product of protein metabolic modifications, dityrosine network (DTN) formation, is an important element in the host–microbe homeostasis. DTN is dependent upon a dual oxidase (*duox*), which is a member of the NADPH oxidase family. *I. scapularis* encodes such oxidase and peroxidase involved in DTN formation. It has been shown that silencing of *duox* or the peroxidase impairs DTN formation, which results in activation of tick immunity and reduced *B. burgdorferi* colonization. Knockdown of *duox* is also associated with increased nitric oxide synthase activity, which is responsible for production of reactive nitrogen species that are considered additional protective defense that ticks use to control pathogens, including *B. burgdorferi* (Yang et al., 2014; Kitsou and Pal, 2018; Kurokawa et al., 2020). Furthermore, during infection of tick cells with Langat virus (a naturally attenuated member of the TBEV complex), *Ixodes scapularis* tick GST has been shown to increase the ability to generate oxidative stress, which affects cell survival and the viability of the tick cell line. It has also been shown that Langat virus can use tick peroxyredoxins to facilitate replication in host cells. This underlines the importance of coping mechanisms with oxidative stress not only at the level of the host organism but also for pathogen transmission and the viral pathogenesis itself (Kusakisako et al., 2020; Hernandez et al., 2021). **Table 1** shows tick redox metabolism changes associated with tick-borne pathogens.

**Table 1:** Tick redox metabolism changes associated with tick-borne pathogen infection.

Tick species	Pathogen	Effect of the Pathogen on Tick Metabolism	References
*Rhipicephalus microplus*	*Anaplasma marginale*	Downregulation of genes encoding ROS-generating enzymes (dual oxidase and endoplasmic reticulum oxidase) Upregulation of antioxidant enzymes: superoxide dismutase, catalase, gluthatione peroxidase, glutathione S-transferase (GST), thioredoxin, thioredoxin reductase, and peroxiredoxin	Kalil et al., 2017
		Upregulation of GST and cytochrome *c* oxidase sub III (COXIII) genes; knockdown of the *Rhipicephalus microplus* cytochrome c oxidase subunit III gene is associated with an inability of *Anaplasma marginale* transmission	Bifano et al., 2014
*Ixodes scapularis* (tick embryonic cell line IDE8)	*Anaplasma marginale*	Overexpression of GST, cytochrome *c* oxidase subunit II, and selenoprotein	de la Fuente et al., 2007
*Ixodes scapularis*	*Anaplasma phagocytophilum*	Modulation of the levels of genes and proteins of hemoglobinolytic enzymes affecting host hemoglobin levels in tick tissues, leading to reduction of the antimicrobial oxidative burden caused by reactive oxygen species (ROS) generated after heme release	Villar et al., 2016
		Increase of mitochondrial ROS production in tick cells, inhibition of alternative ROS production pathways and apoptosis	Alberdi et al., 2019
*Ixodes ricinus*	*Borrelia burgdorferi*	Induction of thioredoxin peroxidases and GST	Rudenko et al., 2005
*Ixodes scapularis*	*Borrelia burgdorferi*	Expression of dual oxidase (Duox) during early tick feeding, induction of nitric oxide synthase, and induction of gut peroxidases in presence of spirochetes; impaired dityrosine network in Duox knockdown or in specific peroxidase knockdown ticks reduces levels of *B. burgdorferi* persistence within ticks	Yang et al., 2014
		Different effects depending on the *B. burgdorferi* strain: Bre-13 infection causes upregulation of GST in tick salivary glands; downregulation of GST in B31 infection, upregulation of protein disulfide isomerase in PBi, B31, and N40 infection, and upregulation of heme lipoprotein precursor in PBi and B-31 infection	Cotté et al., 2014
*Ixodes scapularis*	Langat virus	Upregulation of GST	Hernandez et al., 2021
*Amblyomma maculatum*	*Rickettsia parkeri*	Upregulation of selenogenes in tick midgut tissues and salivary glands	Budachetri et al., 2018
		Increased expression of catalase gene in midgut, salivary glands, and ovarian tissues	Budachetri et al., 2017b

### Role of Tick Saliva in Redox Balance

After tick attachment, tick saliva stimulates a local cutaneous immune response at the tick feeding site. To prevent the invasion of the microorganisms, the vertebrate host’s immune system activates phagocytes, including neutrophils, monocytes, macrophages, and eosinophils, which produce abundant amounts of superoxide anions, leading to higher oxidative stress in ticks. Tick saliva is also a rich source of bioactive molecules, including proteins, peptides, and lipid derivatives. Some of these molecules can modulate the innate and adaptive host immune responses and facilitate the transmission of the pathogen (Aounallah et al., 2020; Karim et al., 2021). Dopamine is known to be the most potent regulator of tick salivary fluid secretion. It is a neurotransmitter that additionally affects the release of AA arachidonic acid, which is then converted into prostaglandins. Furthermore, tick saliva is characterized by extremely high levels of prostaglandins, which have additional influence the host’s metabolism (Karim and Adamson, 2012).

It has been demonstrated that tick salivary secretions are essential for pathogen transmission. The salivary gland extract from *Ixodes ricinus* ticks has been shown to inhibit the killing of *Borrelia afzelii* spirochetes by murine macrophages. It reduces generation of superoxide anions and nitric oxide and thus may facilitate the transmission of the spirochete (Kuthejlová et al., 2001). Accordingly, data suggest that *B. burgdorferi* infection modifies protein content in tick saliva so that it promotes the survival of the pathogen. The spirochete suppresses the copper/zinc SOD that generates hydrogen peroxide, which is toxic to *B. burgdorferi*, while it enhances activity of cytoprotective enzymes, such as catalase, thioredoxin, and pyruvate kinase (Kim et al., 2021). Furthermore, there is a GSH-Px homolog, named salp25D that is expressed in both unfed and fed salivary glands of nymphs. Salp25D is an antioxidant salivary protein from *I. scapularis*, which detoxifies ROS at the vector–pathogen–host interface providing a survival advantage to *B. burgdorferi* at the tick feeding site (Narasimhan et al., 2007). It is possibly involved in acquisition of *B. burgdorferi* by ticks as silencing of Salp25D expression in salivary glands impairs acquisition of *B. burgdorferi* (Narasimhan et al., 2007; Fogaça et al., 2021). Another tick salivary protein involved in the pathogenesis of tick-borne diseases is sialostatin L2, which has been found to inhibit inflammation during *Anaplasma phagocytophilum* infection. It inhibits ROS production by wild-type macrophages during *A. phagocytophilum* stimulation, and it has been implicated that sialostatin L2 regulates ROS production through NADPH-dependent and, probably, independent pathways. Furthermore, *A. phagocytophilum* actively inhibits NADPH oxidase and, consequently, ROS production in neutrophils (Chen G. et al., 2014). Interestingly, it was demonstrated that longistatin, another molecule that is secreted in the saliva of ticks (particularly *Haemaphysalis longicornis* tick), binds the receptor for advanced glycated end products, through which it mediates the host immune response suppressing the tick bite-associated inflammation. Treatment of human umbilical vein endothelial cells with longistatin prior to stimulation attenuates cellular oxidative stress and prevents inflammation by reduction of NF-κB translocation, leading to reduced adhesion molecule and cytokine production (Anisuzzaman et al., 2015). Moreover, it has been indicated that immunoregulatory peptides from the salivary glands of *Hyalomma asiaticum*, hyalomin-A and hyalomin-B, limit the host’s inflammatory response by cytokine secretion inhibition and ROS neutralization (Wu et al., 2010). Altogether, these findings support the importance of redox balance in tick saliva, which seems to be essential for pathogen survival and transmission.

## The Role of Oxidative Stress and Inflammation in Tick-Borne Diseases

### Lyme Disease

LD caused by *Borrelia burgdorferi* s.l. spirochetes is the most prevalent tick-borne infection in humans (Steere et al., 2016; Boulanger et al., 2019). It can be divided into three stages: early localized (EM and borrelial lymphoma), early disseminated (multiple EM, neuroborreliosis, Lyme arthritis, carditis, and other organ manifestations), and late (neuroborreliosis, Lyme arthritis, and acrodermatitis chronic atrophicans) (Cardenas-de la Garza et al., 2018).

The most common and early symptom of *B. burgdorferi* infection is a locally migrating skin rash called EM, which, if left untreated, can cause inflammatory complications in the joints, heart, or nervous system (Bernard et al., 2020). The infection begins to develop around the tick bite site a few days to several weeks after infection. The transcriptomic profile of the skin biopsies of patients with EM shows induction of chemokines, cytokines, Toll-like receptors, antimicrobial peptides, markers of monocytoid cells activation markers, and numerous IFN-induced genes, which play a key role in avoiding resistance to some pathogens by driving T-cell regulatory differentiation (Marques et al., 2017). It is believed that T lymphocytes, activated dendritic cells and pro-inflammatory cells, as well as IL-6 and IFN-γ cytokines play a dominant role in the development of the disease (Steere et al., 2016), because in response to the transmission of *B. burgdorferi*, basic epidermal cells (keratinocytes) secrete inflammatory cytokines and antimicrobial peptides (Bigelmayr et al., 2019). However, the basic cells of the dermis, fibroblasts, react to *B. burgdorferi* with the secretion of chemokines, pro-inflammatory cytokines, IFN (types I, II, or III), and matrix metalloproteinases (MMPs) through NF-κB–dependent mechanisms and therefore MMPs are believed to promote the spread of bacterium (Schramm et al., 2012). In addition, it has been found that, in response to *B. burgdorferi* infection, both macrophages and dendritic cells increase the production of the anti-inflammatory IL-10, which is capable of blocking many immune functions of antigen presenting cells, which may be critical in controlling infection, including phagocytosis and induction of ROS and NO, as well as secretion of inflammatory cytokines/chemokines (Chung et al., 2013). Moreover, EM is accompanied by a decrease in the activity of the main antioxidant enzymes such as SOD and GSH-Px and the level of GSH observed in the blood plasma, which results in tendency into oxidative stress and a significant increase in lipid peroxidation estimated from the level of MDA (Pancewicz et al., 2001). It was also indicated that changes in GSH level may persist for several months after the onset of infection (Kerstholt et al., 2018). The important role of GSH in *B. burgdorferi* infection was also supported by other studies (Casselli et al., 2017), which found that two GST genes (GSTT1 and GSTM1) are among the most affected genes after bacterial exposure in primary human astrocytes, whereas GST catalysis interaction of GSH with lipid hydroperoxide also detoxifies the lipid peroxidation end product (Sharma et al., 2004). In addition, a genome-wide study showed a genetic variant in MAT2B associated with *B. burgdorferi* seropositivity (Hammer et al., 2015). MAT2B encodes the regulatory subunit of MAT2A, one of the enzymes preceding GSH metabolism. However, *B. burgdorferi* infection affects the expression of the MAT2A gene in the peripheral blood mononuclear cells of patients with EM. This suggests that the metabolism of GSH may also play a role in the production of antibodies to the bacterium. It has been found, however, that treatment of patients with beta-lactam antibiotics normalizes the level of GSH but does not change the activity of antioxidant enzymes, which does not favor the reduction of oxidative stress accompanying the infection (Pancewicz et al., 2001).

**Figure 3:**
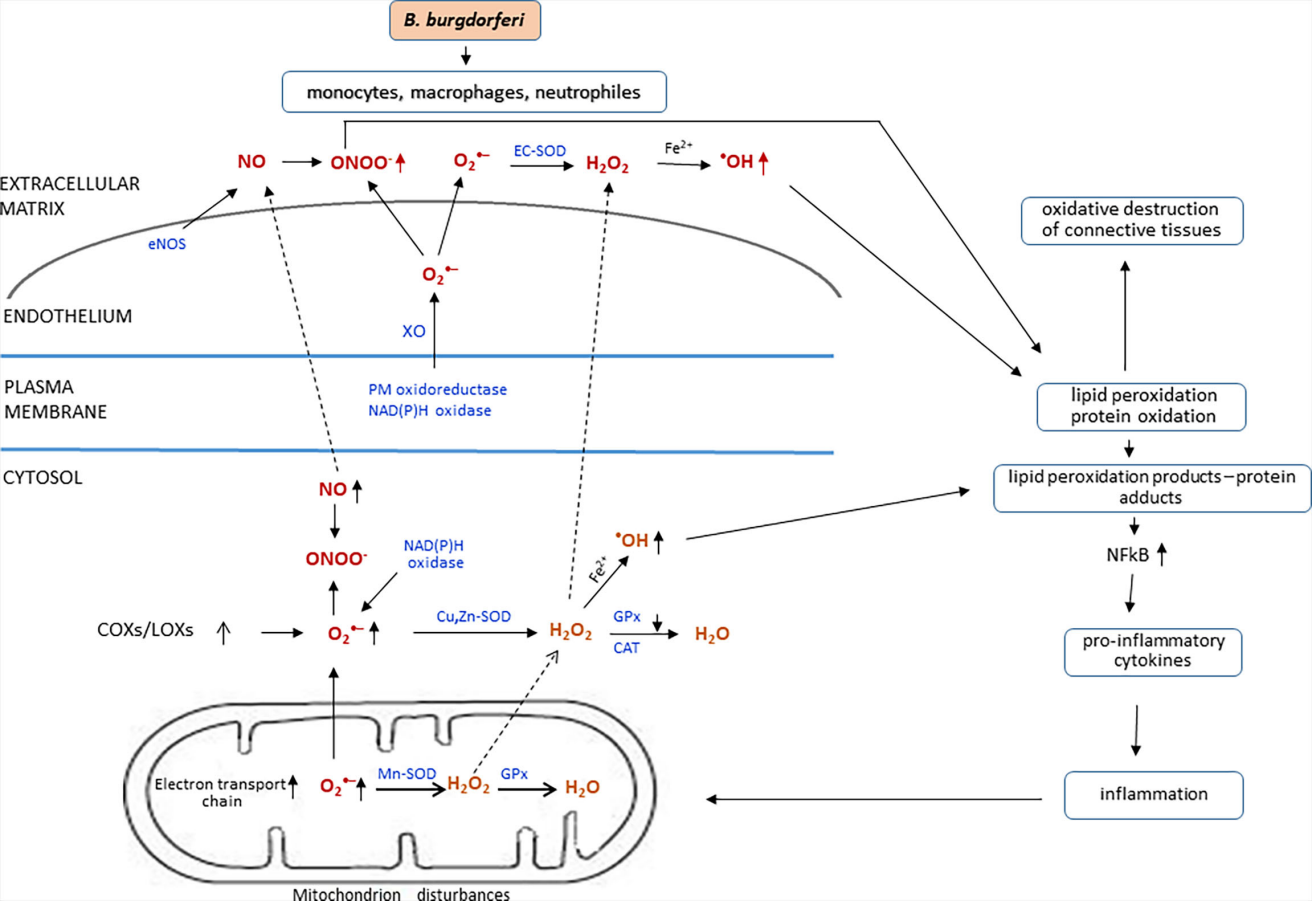
Redox imbalance and metabolic effects of *B*. *burgdorferi*.

Currently, it is suggested that the host organism’s response to infection may be two-phase. Initially, there may be an increase in intracellular GSH concentration and a decrease in ROS production in response to bacterial invasion, whereas, after prolonged exposure, GSH level is decreased and ROS generation is increased, and, consequently, oxidative stress is generated (**Figure 3**) (Kerstholt et al., 2020). However, *in vitro* studies on monocytes stimulated with *B. burgdorferi*, which can be compared with the acute phase of bacterial activity, showed an over 10-fold increase in intracellular concentration of GSH (Kerstholt et al., 2018), the primary non-enzymatic antioxidant in cells. The above observations are essential because ROS are an important element of the host’s defense against pathogens, and in addition, a reduced level of ROS may increase the host’s susceptibility to other pathogens, whereas GSH, regardless of its antioxidant properties, also affects cell signaling and inflammation. However, the long-term effects of *B. burgdorferi* invasion indicates reduction of the level of calcium ions by lowering the level of CD38 glycoprotein, which, in combination with inflammation, leads to mitochondrial dysfunction manifested by an additional increase in ROS production and reduced antioxidant capacity in patients infected with *B. burgdorferi* (Peacock et al., 2015). Later, LD is accompanied by an increased generation of ROS as demonstrated in Browicz–Kupffer cells, neutrophils, and monocytes with the release of superoxide anion radicals, hydrogen peroxide, and nitric oxide to the extracellular space and the formation of peroxynitrites in their interaction, indicating the beginning of oxidative degradation and destruction of connective tissue (Guerau-de-Arellano et al., 2005; Ratajczak-Wrona et al., 2013; Peacock et al., 2015). Moreover, further exacerbation of *B. burgdorferi* infection induces expression of β2/CD18, which mediates migration, adhesion, and activation of neutrophils and other leukocytes to the vascular endothelium, leading to the development of Lyme arthritis or neuroborreliosis. *B. burgdorferi*, demonstrating pro-oxidative activity, affects the metabolic and signaling pathways of the host cells, including passive adsorption of host cysteine, leading to a reduction in the level of GSH (Peacock et al., 2015). Such metabolic modification is essential for the host organism because GSH, regardless of its direct antioxidant activity, also acts as a cofactor of GSH-Px, the main enzyme protecting membrane phospholipids from peroxidation (Peacock et al., 2015). This was confirmed by metabolomic studies, which showed that the greatest impact of *B. burgdorferi* on the human body concerns the metabolism of GSH and arachidonic acid (Kerstholt et al., 2018). The consequence of enhanced oxidative stress and decreased GSH-dependent phospholipid protection, after *B. burgdorferi* infection, is increased lipid peroxidation with an enhanced level of lipid peroxidation products, such as low–molecular weight α,β-unsaturated aldehydes (MDA and 4-HNE) and prostaglandin derivatives (mainly prostaglandins, such as 8-isoPGF2α). However, after antibiotic therapy, the total concentration of 8-isoPGF2α decreases, both in the plasma and urine of patients with Lyme arthritis, which may potentially useful in assessment of the effectiveness of pharmacotherapy (Łuczaj et al., 2015; Łuczaj et al., 2016).

It has been suggested that the host’s response to *B. burgdorferi* infection may be two-phase. In response to bacterial invasion, the redox balance is shifted toward antioxidants, whereas, after prolonged exposure, the generation of reactive oxygen species (ROS) increases, and, as a consequence, oxidative stress is generated. The primary place where ROS are generated in the cell is the mitochondrial respiratory chain, in which, during the flow of electrons, one-electron reduction of oxygen occurs with the formation of a superoxide radical ( $O_{2}^{-}$ ), which, as a result of the action of Mn-dependent superoxide dismutase (Mn-SOD), is dismutated to hydrogen peroxide (H_2_O_2_), which is reduced to water (H_2_O) under the influence of glutathione peroxidase (GPx). Both $O_{2}^{-}$ and H_2_O_2_ pass from the mitochondria into the cytosol. O_2_.− from the mitochondria is supplied with the same radicals, resulting from the action of plasma membrane (PM) oxidoreductase, NAD(P)H oxidase, and xanthine oxidase (XO), as well as cyclooxygenases (COXs), lipoxygenases (LOXs), and in reaction with nitric oxide (NO) produced by endothelial nitric oxide (eNOS) generates peroxynitrate anion (ONOO^−^). On the other hand, as a result of the action of copper- and zinc-dependent superoxide dismutase (Cu, Zn-SOD) in cytosol and extracellular SOD in extracellular matrix, it is dismutated to H_2_O_2_. Hydrogen peroxide is reduced by glutathione reductase (GPx) or catalase (CAT) into water or in Fenton reaction with Fe^2+^into highly reactive hydroxyl radical (.OH), which is generated both in cytosol and extracellular matrix and can cause lipid/protein per/oxidation, which leads oxidative destruction of, e.g., connective tissue. The resulting lipid peroxidation products react with proteins and cause, *inter alia*, increased activity of nuclear factor kappa light-chain enhancer of activated B cells (NF-κB), leading to the biosynthesis of pro-inflammatory cytokines and the development of inflammation.

A similar situation applies to the central nervous system (CNS), which can also be affected by *B. burgdorferi.* CNS is characterized by many mechanisms, promoting high levels of ROS and a relatively low level of antioxidant protection, especially antioxidants preventing lipid peroxidation, including those cooperating with GSH, the level of which is more reduced in the course of neuroborreliosis than in Lyme arthritis (Moniuszko-Malinowska et al., 2016). Therefore, it has been suggested that the neurological symptoms observed during *B. burgdorferi* infection can be related to oxidative stress. Moreover, it was found that *B. burgdorferi*, by generating oxidative stress, stimulates, among others, microglia to produce inflammatory mediators such as IL-6, TNF-α, and PGE2, and this phenomenon plays an important role in the development of LD (Rasley et al., 2002). It is known that the mutual driving of the development of inflammation and oxidative stress is the result of the interaction of two transcription factors: Nrf2 regulating oxidative stress through the biosynthesis of antioxidants and NF-κB modifying inflammation by modulating the generation of pro-inflammatory cytokines (Wardyn et al., 2015). The brain tissue is also characterized by a high content of phospholipid structures susceptible to peroxidation, the increased intensity of which was observed in the cerebrospinal fluid of patients with neuroborreliosis, assessed as products of oxidative cyclization (isoprostanes/neuroprostanes) and oxidative fragmentation (α,β-unsaturated reactive aldehydes). This is accompanied by a reduction in the level of PUFAs (arachidonic and docosahexaenoic acid) observed in the plasma of patients with neuroborreliosis (Moniuszko-Malinowska et al., 2016). Modifications of the phospholipids of the brain structures with reactive aldehydes formation, which form adducts with proteins (Gęgotek and Skrzydlewska, 2019), can increase the permeability of the blood–brain barrier. This allows the release of, e.g., products of lipid peroxidation into the blood, which, due to their reactivity, may result in a systemic response. As a consequence, in the course of LD, the activity of enzymatic antioxidants and the level of non-enzymatic components of the antioxidant system decrease, and, consequently, the concentration of lipid peroxidation products in the plasma and urine of patients increases (Łuczaj et al., 2017). Because of the unique reactivity of α,β-unsaturated aldehydes generated during lipid peroxidation, including the most frequently evaluated 4-HNE in plasma, an increased concentration of 4-HNE–modified proteins in the plasma of patients with neuroborreliosis has also been demonstrated, which indicates a different, not directly oxidative, but resulting from the modification of the structure and, consequently, the function of proteins (Moniuszko-Malinowska et al., 2016). On the other hand, antibiotic therapy not only reduces the level of lipid peroxidation products, which indicates the possibility of their use as biomarkers of the disease, but also may be useful in assessing the effectiveness of the treatment of neuroborreliosis (Moniuszko-Malinowska et al., 2016).

**Figure 4:**
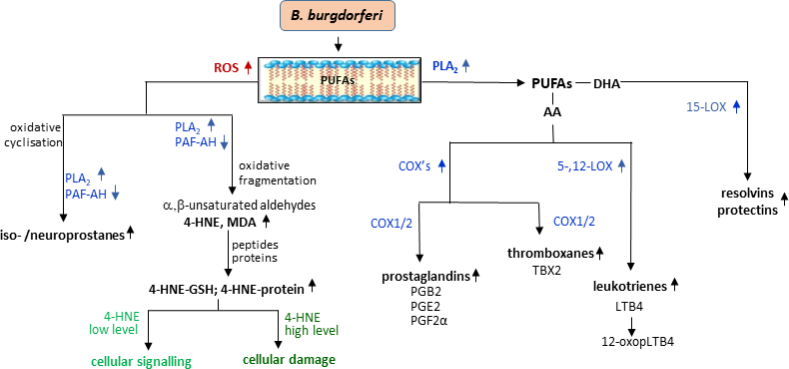
Changes in phospholipid metabolism with lipid mediators generation during *B*. *burgdorferi* infection.

The oxidative stress accompanying the development of LD promotes not only ROS-dependent modifications of phospholipids but also by increasing the activity of enzymes involved in the metabolism of phospholipids, leading to enzyme-dependent increased generation of lipid mediators (**Figure 4**) (Pratt and Brown, 2014; Brown and Dennis, 2017). The most widely studied arachidonic acid (20:4) is oxidized by two major metabolic pathways including COXs (COX-1 and COX-2) catalyzing the production of pro- and anti-inflammatory prostaglandins and thromboxanes as well as with the participation of LOXs, producing pro-inflammatory leukotrienes and pro-resolving lipoxins (Tam, 2013; Brown and Dennis, 2017). In addition to arachidonic acid, omega-3 fatty acids, DHA and EPA, are metabolized by the same enzymes for the production of other bioactive lipid metabolites such as protectins and resolvins (Serhan and Petasis, 2011). The compounds formed as a result of enzymatic oxidation play a role in both induction and resolution of inflammation, whereas disorders of the latter may cause prolonged inflammation and, as a consequence, more severe course of the LD and complications such as Lyme arthritis (Serhan et al., 2008). Moreover, it is known that not all inflammatory cells are characterized by the production of products *via* above pathways. In addition, different cells show considerable variability in the production of specific metabolites, e.g., macrophages tend to produce particularly high levels of prostaglandin E2, whereas neutrophils tend to produce large amounts of LTB4 leukotrienes (Kihara et al., 2014) In response to *B. burgdorferi* infection, there is an increased expression of constitutive COX-1 in lymphocytes B, which is responsible for the synthesis of prostaglandins and thromboxane BX (Blaho et al., 2009b). In the development of LD, an increase in the expression and activity of COX-1 and COX-2 is usually accompanied by an increase in the level of prostaglandins, including PGE2 and PGF2α as well as TXB2 and expression of prostaglandin receptor, EP2 (Blaho et al., 2009b). On the other hand, blocking both COX isoenzymes (COX-1 and COX-2) reduces the concentration of prostaglandins in murine B cells and the severity of arthritis in a mice model of LD (Blaho et al., 2009b). A similar situation was observed in COX-1^−/−^ mice infected with *B. burgdorferi* (Jarosz and Badawi, 2019). Furthermore, the expression of COX2, which is usually undetectable in normal tissues, is elevated in murine LA models and in microglial cells in a model of neuroborreliosis (Jarosz and Badawi, 2019; Ramesh et al., 2017). However, despite the reported lower levels of lipid mediators such as PGD2, PGE2, LTC4, LTE4, and 5-HETE in COX-2^−/−^ and 5-LOX^−/−^, mice do not recover (Jarosz and Badawi, 2019). It is also believed that prostaglandin E2 may have pro-inflammatory or anti-inflammatory effects depending on which receptor it activates (Shinomiya et al., 2001). Such situation corresponds to an increase in PGE2 levels in the joints during induction and resolution of Lyme arthritis as determined by lipidomic studies (Blaho et al., 2009a). This suggests that both metabolic pathways responsible for generation pro-inflammatory mediators also play a role in reducing inflammation in Lyme arthritis.

The metabolism of arachidonic acid is believed to be one of the major pathways during *B. burgdorferi* infection. This is evidenced by the increase in the level of prostaglandin PGB2, a product of PGA2 isomerization, which is formed as a result of enzymatic dehydration of PGE2, an eicosanoid associated with pro-inflammatory effects (Buczynski et al., 2009; Fitzgerald et al., 2020). Elevated PGB2 levels in the early stage of LD may reflect elevated PGE2 levels in the later stage of the disease. Another eicosanoid that is elevated in LD is 12-oxo-LTB4, which is a degradation product of LTB4, an important pro-inflammatory lipid mediator and chemotactic factor (Yokomizo et al., 1993; Fitzgerald et al., 2020). In addition, LTB4 induces the release of potent antimicrobial compounds, such as α-defensin and cathelicidin, and lysosomal enzymes but also activates NADPH oxidase and NO production to enhance bacterial killing. LTB4 transmits signals through two G protein–coupled receptors (BLTs), including the high-affinity receptor (BLT1) (Yokomizo, 2015). The importance of this signaling is evidenced by the fact that *B. burgdorferi*–infected BLT1^−/−^ mice are characterized by persistent Lyme arthritis, with later increased numbers of neutrophils in the joints (Hilliard and Brown, 2019). However, 12-oxo-LTB4 is associated with anti-inflammatory mechanisms (Wainwright and Powell, 1991; Yokomizo et al., 1993). Increasing the level of both of the above eicosanoids (PGB2 and 12-oxo-LTB4) in early LD may result from an attempt to suppress the inflammatory response by the host organism (Yokomizo et al., 1993; Fitzgerald et al., 2020). Because the metabolic changes of fatty acids lead to the formation of both pro-inflammatory and anti-inflammatory substances in one metabolic sequence, for both diagnostic and therapeutic purposes, it is important to determine the level of finally formed compounds and, consequently, to determine the dominant form pro-inflammatory or anti-inflammatory.

The oxidative stress accompanying LD promotes both direct ROS-dependent modifications of phospholipids and also enzyme-dependent changes in phospholipid metabolism. The action of ROS on phospholipids results in the formation of oxidative cyclization products, which are released into the cytosol by phospholipase A_2_ (PLA2) and platelet-activating factor acetylhydrolase (PAF-AH). On the other hand, the action of ROS on fatty acids released by PLA2 leads to the formation of electrophilic oxidative fragmentation products, e.g., malondialdehyde (MDA)/4-hydroxynonenal (4-HNE), which react with the nucleophilic elements of peptides and proteins, modifying their structure and functions depending on the concentration. In addition, free fatty acids (PUFAs) are metabolized by cyclooxygenases (COX1/2) and lipoxygenases (5,12-LOX and 15-LOX), which results in the formation of pro- or anti-inflammatory lipid mediators including pro-and anti-inflammatory prostaglandins (PG) and thromboxanes (TBX) and with the participation of lipoxygenases (LOXs), producing pro-inflammatory leukotrienes (LTs) and anti-inflammatory resolvins and protectins, which play a role in both induction and resolution of inflammation.

It is known, however, that as a result of LOXs-dependent phospholipid metabolism, which is a response to an infection, resolvins and protectins with anti-inflammatory profile are generated (Dennis and Norris, 2015). As a result of the metabolism of DHA by 15-LOX, D-series resolvins and protectin, NPD1, are generated, which show anti-inflammatory and protective effects on the nervous system. Like resolvins, NPD1 exerts an inhibitory effect on the PMNL infiltration through autocrine or paracrine effects, reducing the expression of cytokines (Dennis and Norris, 2015). Protectin NPD1 is also found in the joints of mice after resolution of LD (Blaho et al., 2009a). The influence of pathogens on the host’s organism also depends on the genetic variability of the host. On the basis of lipidomic studies, it was suggested that DBA mice, due to high levels of protectin 1 (PD1), resolvin D1 (RvD1), hepoxylin A3 (HXA3), PGE2, and 15-keto PGE2, are resistant to the development of LD after *B. burgdorferi* infection (Blaho et al., 2009a). In contrast, the response of C3H mice is characterized by prolonged inflammation and delayed regeneration, which is associated with high levels of LTB4 (Blaho et al., 2009a). This suggests a defect in the synthesis of anti-inflammatory and pro-inflammatory mediators in *B. burgdorferi–*infected C3H mice. Therefore, it is suggested that, when analyzing the host’s response, one should take into account not only the type of pathogen but also the individual variability of the host’s susceptibility to oxidative and inflammatory conditions induced by the pathogen.

Regardless of the hydrolysis of phospholipids by PLA2, their metabolism, especially with respect to phosphatidylcholine and phosphatidylethanolamines, also involves PLD contributing to the formation of N-acylethanolamines, including anandamide and other derivatives belonging to the group of endocannabinoids. N-acylethanolamines have been identified in early LD in animal tissues, including the skin (Dalle Carbonare et al., 2008; Iannotti et al., 2016). Significantly elevated levels of N-acylethanolamines in the serum of patients with STARI (southern tick-associated rash disease) were observed. These compounds formed in response to inflammation have anti-inflammatory properties as PPAR-α agonists or enhance the activity of anandamide (Lo Verme et al., 2005; Di Marzo et al., 2001). N-acylethanolamine can be metabolized by alcohol dehydrogenase to N-acylglycine derivatives and further degraded to primary fatty acid amide (PFAM). It has been suggested that the increased levels of NAE and PFAM may be due to decreased fatty acid amide hydrolase activity, which degrades the above compounds (Cravatt et al., 1996). The known anti-inflammatory effect of NAE also suggests the possibility that these metabolites are partly responsible for the relief of symptoms associated with infection (Wormser et al., 2005).

### Viral Infections

Another tick-borne pathogen is TBEV, a member of the *Flaviviridae* family. This virus is the etiological agent of TBE, a disease of the central nervous system that is endemic in Central and Eastern Europe, Siberia, the Far East Russia, northern China, and Japan (Amicizia et al., 2013; Bogovic and Strle, 2015). TBEV occurs as Far Eastern, Siberian, and European serotypes and is transmitted mainly through a bite by the *Ixodes* spp. ticks, but infection can also occur through the consumption of unpasteurized milk from infected livestock (Bogovic and Strle, 2015).

The disease caused by European TBEV subtype is typically characterized by a biphasic course. In the first viraemic phase, predominating symptoms include fever, fatigue, headache, and arthralgia. Neurological manifestations are the hallmark of the second phase, with a clinical spectrum ranging from mild meningitis to severe encephalitis, which may be accompanied by myelitis and acute flaccid paralysis. TBE presents as meningitis in about 50%, as meningoencephalitis in 40%, and as meningoencephalomyelitis in 10% of patients. TBE is fatal in about 1%–2% of patients (Kaiser, 2008; Lindquist and Vapalahti, 2008). Up to half of the patients report lingering symptoms 6–12 months post-TBE, with severe neurological impairment in 30% of the patients (Łuczaj et al., 2016).

**Figure 5:**
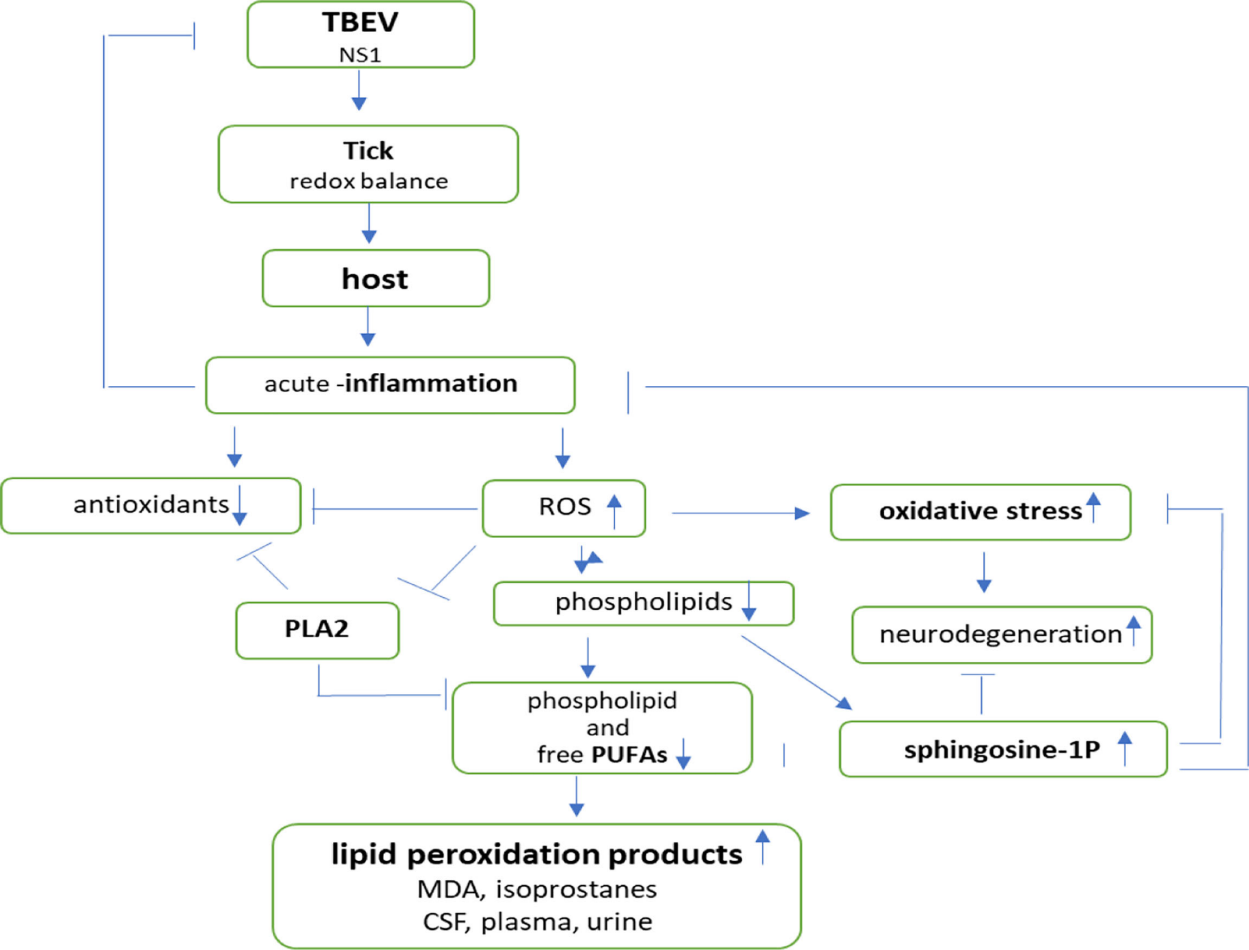
Effects of pathogen–vector–host interactions in TBE infection.

In arthropod-borne viral infections, oxidative stress plays an important role in pathogen–vector–host interactions (**Figure 5**). The maintenance of the redox balance by vectors is believed to be an important part of pathogen transmission (Kusakisako et al., 2020; Hernandez et al., 2021). It was found that NS1 protein is distinctive of flaviviruses and is required for RNA replication and infectious particles production. It is known that the expression of the NS1 protein of the TBEV leads to increased production of ROS, which is compensated by the activation of one of the most important antioxidant mechanisms—the Nrf2/ARE pathway (Kuzmenko et al., 2016).

In addition, TBEV is known to disrupt many metabolic pathways in the host organism, including those related to redox balance. TBEV infection leads initially to a pro-inflammatory and then anti-inflammatory response. Although an acute inflammatory response is a prerequisite for initiating pathogen elimination, pro- and anti-inflammatory processes are essential as they prevent excessive pathological damage to the host organism (Du et al., 2021). Increased ROS production and decreased activity of antioxidant enzymes (SOD, GSH-Px, and GSSG-R) enhance lipid peroxidation (Łuczaj et al., 2016). Moreover, a decrease in glutathione peroxidase activity in the plasma of patients with TBE indicates a reduced protection of brain lipid structures against oxidative damages. In addition, the reduced activity of PLA2 in TBE additionally inhibits the action of GSH-Px and enhances lipid peroxidation (Łuczaj et al., 2016). As a consequence during TBE course, there is a decreasing tendency in the levels of free and phospholipid linoleic acid, arachidonic acid, and docosahexaenoic acid in plasma, which indicates intensification of their metabolism. In patients with TBE, an increase in the concentration of lipid peroxidation products, including reactive aldehydes such as MDA, acrolein, crotonaldehyde, and 4-HHE in the cerebrospinal fluid; 4-HNE, MDA, and acrolein in plasma; and 4-HHE, 4-ONE, MDA, and acrolein in the urine of patients, was observed. Furthermore, the concentration of F2-isoprostanes and neuroprostanes, which are prostaglandin-like compounds that are considered relatively stable markers of lipid peroxidation, is also significantly increased in CSF and plasma of patients with TBE (Łuczaj et al., 2016). F2-isoprostanes are formed mainly by the non-enzymatic peroxidation of arachidonic acid, whereas neuroprostanes are derived from docosahexaenoic acid, an important structural component of neuronal membranes. Neurons are particularly sensitive to oxidative damage as they have weak antioxidant defenses and contain plenty of unsaturated fatty acids that are prone to oxidation. Furthermore, neuronal cells accumulate metal ions, which are able to catalyze oxidative species formation reactions (Chen CY et al., 2014). Literature data indicate that F2-isoprostanes and neuroprostanes are significantly increased and can be utilized as biomarkers of oxidative stress in some of neurodegenerative disorders as well as in the course of TBE (Lima et al., 2012) According to several studies, neurodegenerative process is also present in the course of TBE and persists even after clinical recovery of patients (Czupryna et al., 2018; Czupryna et al., 2019). Brain response to oxidative stress-mediated neurodegeneration is a very complex process involving all brain structures. Microglial cells are main mediators of neuroinflammation. In response to oxidative stress, microglial cells transform into activated microglia. ROS produced by microglia and the surrounding environment not only impact neurons but also modulate microglial activity. Chronic activation of microglia leads to release of potentially toxic molecules, such as proinflammatory cytokines, MPPs, ROS/RNS, proteinases, prostaglandin E2, complement proteins, and growth factors, and, consequently, may lead to neuronal damages (Miller et al., 2014; Rojo et al., 2014).

Alterations in phospholipids, sphingomyelins, and triglycerides metabolism may partly explain the clinical picture of TBEV infection, which includes fever, nausea, and meningoencephalitis (Sun et al., 2021). TBE also affects the level of acylcarnitine, which is essential for lipid metabolism, particularly fatty acid β-oxidation (Kępka et al., 2017; Du et al., 2021). It is suspected that alterations in serum acylcarnitine levels may reflect the mitochondrial dysfunction associated with disturbed transport of fatty acids (Du et al., 2021). Furthermore, TBE is associated with increased concentration of S1P, a product of sphingomyelin metabolism, in cerebrospinal fluid. S1P is a signaling molecule that is involved in inflammation and immune reactions and has a pivotal role in neuronal differentiation, survival, and excitability. It is involved in neuronal repair and is known to decrease oxidative stress in neuronal cells (Kułakowska et al., 2014; Chung et al., 2020). During febrile stage of TBE, triacylglycerol expression is observed (Du et al., 2021). An untargeted metabolomics analysis of serum shows that phospholipids may be used to distinguish patients with TBE from healthy controls. Fatty acid biosynthesis and glycerophospholipid metabolism pathway metabolites, e.g., lysophosphatidylcholine (18:1 and 15:0), 3-O-sulfogalactosylceramide, and PE(P-16:0e/0:0) (a phospho-ether lipid), were significantly different in patients with TBE (Zhang et al., 2021).

### Other Tick-Borne Diseases

Ticks are also vectors of other pathogens, including *Anaplasma phagocytophilum*, the causative agent of HGA, *Babesia microti* causing babesiosis, and bacteria belonging to *Rickettsiaceae family* including the spotted fever group (SFG) and the typhus group agents (Blanton, 2019; Boulanger et al., 2019). *A. phagocytophilum* is an obligate intracellular bacterium, which replicates in neutrophils. The infection and colonization of ticks with *A. phagocytophilum* first occur in midgut cells and subsequently hemocytes and salivary glands, from where the pathogen is transmitted during feeding. To establish infection, *A. phagocytophilum* induces complex cellular changes and modulates granulocyte major defenses, including oxidative response (Alberdi et al., 2019). The addition of *A. phagocytophilum* to neutrophils (*in vitro*) has been shown to result in little or no detectable levels of ROS, but it induces ROS generation in macrophages, which might explain why these cells may be inappropriate hosts (Santos, 2007; Alberdi et al., 2019). *A. phagocytophilum*–neutrophil interaction leads to a dose-dependent stimulation of NADPH oxidase assembly and degranulation, which suggests that the pathogen has ability of direct superoxide anions detoxification that provides means of protection from oxidative damage. The transcriptional expression of a SOD homolog (*sodB*) has been reported in *A. phagocytophilum.* Furthermore, two other ortholog clusters of proteins potentially involved in oxidative stress response have been identified—a putative heme copper oxidase and a putative flavohemoglobin (Dunning Hotopp et al., 2006; Santos, 2007) *A. phagocytophilum* significantly reduces NADPH oxidase subunits gp91(phox) and p22(phox) levels in the phagosomal membrane. It has been suggested that the suppression of ROS production in human cell line model of *A. phagocytophilum* infection is regulated through pathogen effector Ankyrin A (AnkA)–dependent downregulation of NADPH oxidase (Alberdi et al., 2019). AnkA is a member of family of proteins called nucleomodulins, which are involved in host gene expression control at the epigenetic level. It is secreted by *A. phagocytophilum* in infected neutrophils through the bacterial type IV secretion system (T4SS). Upon entering the granulocyte nucleus, AnkA binds stretches of AT-rich nuclear DNA altering transcription of antimicrobial defense genes, including downregulation of *CYBB* that codes NADPH oxidase 2 (NOX2). It is an enzyme responsible for production of ROS, which are an essential part of neutrophil immune response against intracellular pathogens (Santos, 2007; Cabezas-Cruz et al., 2017).

Anaplasmosis infection in animals (caused by different species than *A. phagocytophilum*) has been shown to induce oxidative stress in the organism (De et al., 2012). In cattle infected with *Anaplasma marginale*, the antioxidant capacity of erythrocytes is diminished due to the reduction of SOD activity and the level of glutathione, which results in oxidative stress formation and increased lipid peroxidation (De et al., 2012; Esmaeilnejad et al., 2018). It has been suggested that this is due to the high content of PUFAs in the erythrocyte membrane, which favors the peroxidation process. Similar observations regarding oxidative stress with the decrease in the activity of glutathione peroxidase and increased erythrocyte lipid peroxidation estimated by MDA were made in *Babesia bigemina* infection in calves (De et al., 2012, Salem et al., 2016). It has also been demonstrated that *Babesia ovis* infection decreases total antioxidant activity, leading to increase of oxidative stress markers and DNA damages in blood samples of infected goats (Kucukkurt et al., 2014). Similarly to other aerobic parasites, *Babesia* lives in an oxygen-rich environment in its mammalian host (primarily during the erythrocytic stage), resulting in exposure to the toxic effects of ROS. *Babesia* is one of the examples of the importance of the parasite’s antioxidant system for invasion and proliferation inside erythrocytes (Bosch et al., 2015). Furthermore, it has been shown that *B. divergens* leads to significant redox imbalance in hepatic tissues of Mongolian gerbils. It has been also demonstrated that the pathogen causes hepatic tissue damage *via* oxidative stress. The study showed decreased GSH and CAT concentration, increased MDA level, alteration of the nitrite/nitrate levels, and a decrease in the total antioxidant capacity in hepatic tissue (Dkhil et al., 2013). Increased MDA levels have also been reported in *B. gibsoni* infections and in co-infections of *Ehrlichia canis* and *B. gibsoni* in dogs (Bosch et al., 2015). It has been also shown that oxidative stress may be one of the mechanisms, leading to anemia in dogs with babesiosis and antioxidant biomarkers, copper, and zinc concentrations can be used as indicators of disease severity and prognostic markers (Teodorowski et al., 2021). The abovementioned results indicate that the infection of animals with *Anaplasma* spp. and *Babesia* spp. induces oxidative stress. There is limited number of studies regarding the redox imbalance in HGA and human babesiosis; however, the above data may suggest that the human body may respond in a similar way to infection with these pathogens.

Another intracellular tick-borne pathogen is *Rickettsia rickettsii*, the etiological agent of Rocky Mountain spotted fever. It has been demonstrated that human endothelial cells infected with this pathogen accumulate superoxide anion ( $O_{2}^{-}$ ) and extracellular H_2_O_2_ and undergo oxidative stress. Exposure to *R. ricketsii* results in induction of superoxide and SOD in endothelial cells (Santucci et al., 1992). Furthermore, lipid peroxidation seems to be an important mechanism in the endothelial cell injury caused by *R. ricketsii* (Silverman and Santucci, 1988). Oxidative stress induces higher expression of HO-1, a heme catabolism enzyme with antioxidant properties. Bilirubin and carbon monoxide are end products of HO-1 activity that decrease oxidative stress, reduce vascular constriction, attenuate inflammation, and inhibit apoptosis. Heme-HO system also participates in the synthesis and secretion of prostaglandins through regulation of vascular COX activity. During rickettsial infections, induction of COX-2 and increased secretion of PGE2 and PGI2 with the biphasic increase in COX-2 expression resembling NF-κB activation are observed. The induction of COX-2 together with prostaglandin release possibly contributes to increased vascular permeability during the infection (Sahni et al., 2019). It has been implicated that inducible NO synthase, which synthesizes nitric oxide takes part in control of virulent *Rickettsiae* spp. in diverse cell types. Data suggest that NO-producing macrophages restrict *R. ricketsii* infection, and NO is a potent antirickettsial effector of innate immunity (Fitzsimmons et al., 2021). Furthermore, an antioxidase, *B. microti* thioredoxin reductase (*Bmi* TrxR), has been identified in *B. microti*, which may be important for survival and ROS removal. Inhibition of TrxR is a promising strategy for the control of intracellular pathogens, and it is speculated that development of inhibitors of Bmi TrxR can be useful for the control of *B. microti* (Lu et al., 2021).

## Conclusions

Inflammation and the associated oxidative stress are key elements in the pathogenesis of infectious diseases, including tick-borne diseases. The consequence of redox imbalance is oxidative changes in the structure and function of phospholipids and their metabolites, which affect cellular and systemic metabolism. This disrupts the physiological functioning of the body, which requires pharmacological interventions. Finding a link between patient’s medical condition and changes in metabolism of phospholipids can lead to discovery of unambiguous diagnostic biomarkers. At the same time, such biomarkers would allow to determine the effectiveness of the applied pharmacotherapy.

Notwithstanding the increasingly effective attempts to use phospholipid metabolites as biomarkers of the consequences of changes in the host redox balance for diagnostic purposes, there are also suggestions that, for therapeutic purposes, it seems more rational to modulate the redox metabolism of various microorganisms (i.e., bacteria and viruses) than to manipulate the redox system of the host. This is primarily due to the fact that redox metabolism in microorganisms is reduced only to the basic enzymatic process, and as some microbial enzymes are specific to them, they may be potentially promising molecular targets for the pharmacotherapy of tick-borne diseases.

## Author Contributions

Author contributions to the paper are as follows: Conception: AM-M, ES, SP, and MG; data collection: MG and MD; manuscript draft preparation: MG, MD, ES, SP, and AM-M; figures design and preparation: MG, ES, and MD; review and approval of the final version of the manuscript: MG, ES, MD, SP, and AM-M.

## Funding

The work was supported by the National Science Centre, Poland, under research project 2017/26/E/NZ6/00277.

## Conflict of Interest

The authors declare that the research was conducted in the absence of any commercial or financial relationships that could be construed as a potential conflict of interest.

## Publisher’s Note

All claims expressed in this article are solely those of the authors and do not necessarily represent those of their affiliated organizations, or those of the publisher, the editors and the reviewers. Any product that may be evaluated in this article, or claim that may be made by its manufacturer, is not guaranteed or endorsed by the publisher.
